# Editorial: Cholesterol and Oxysterols as Signal Molecules in Human Pathophysiology and Cancer: Implications for New Therapeutic Strategies

**DOI:** 10.3389/fendo.2019.00732

**Published:** 2019-10-23

**Authors:** Rosa Sirianni, Michihisa Umetani, Vincenzo Pezzi

**Affiliations:** ^1^Department of Pharmacy and Health and Nutritional Science, University of Calabria, Cosenza, Italy; ^2^Center for Nuclear Receptors and Cell Signaling, University of Houston, Houston, TX, United States; ^3^Department of Biology and Biochemistry, College of Natural Sciences and Mathematics, University of Houston, Houston, TX, United States

**Keywords:** cholesterol (chol), oxysterol, cancer, signal molecules, nuclear receptor (NR)

Cholesterol (Chol) is a lipid essential for membrane biogenesis and functionality, cell proliferation, and cell differentiation (1), however its “popularity” comes from hypercholesterolemia, a main risk factor for cardiovascular disease, neurodegeneration, inflammatory bowel disease, and cancer (2, 3). Chol can be the precursor of steroid hormones, bile acids, and other sterol metabolites responsible for a number of specific effects on human physiology (4). Among Chol metabolites, increasing attention is drawn by the family of oxidation products termed oxysterols that have been recently recognized as ligands for nuclear receptors involved in the regulation of cell viability and metabolism (5). Effects promoted by these Chol oxidation products appear to be dependent on their concentration, cell specific, dynamic condition of the cellular and tissue environment (6). For these reasons, the knowledge of molecular mechanisms that regulate the balance of Chol and oxysterol metabolism in different tissues is of crucial importance for understanding the occurrence of several diseases including cancer.

This collection is focused on the role of Chol and/or oxysterols as signaling molecules whose alteration can determine the onset or progression of pathologies. Consequently, modulation of sterol homeostasis represent a promising therapeutic strategy. Yamauchi and Rogers in this collection reviewed the connection between sterol metabolism and atherosclerosis as well as cancers. Their paper focuses on several drugs including those currently used in clinic (i.e., statins to block Chol synthesis and enhance LDL uptake) and those targeting sterol metabolism in preclinical and clinical studies (i.e., drugs targeting SREBPs and SREBP regulators, LXR, ABC transporters, ACAT, and sterol hydroxylases). Chol-free reconstituted or synthetic HDL (sHDL) represent a new reasonable strategy for delivering chemotherapeutic drugs to cancer cells. HDL receptor (Scavenger receptor type B-I; SR-BI), is highly expressed in endocrine cancers, notably due to the high demand for Chol by cancer cells. Binding sHDL loaded with a chemotherapic will deplete Chol while assuring specificity for cancer cells. This attractive approach is reviewed by Morin et al. suggesting the application of sHDLs as endocrine cancer therapeutics.

Patients suffering from hyperlipidemia or metabolic syndrome clearly evidence a link between male reproductive function and Chol homeostasis. Sèdes et al. in this collection highlighted molecular mechanisms involving nuclear receptors LXRs and FXRα and their sterol ligands important for testicular function. Drugs targeting these nuclear receptors could be used in the clinic either to treat male infertility or as new approach for male contraception.

Another interesting aspect is evidenced by Oguro reviewing in this collection recent advances in our understanding of the roles of Chol and its metabolites as signaling molecules in the regulation of hematopoiesis and hematologic malignancies. In particular, Chol metabolism to steroids and oxysterols promotes hematologic cancers, and statins that inhibit *de novo* Chol synthesis have cytotoxic effects in malignant hematopoietic cells.

The therapeutic potential of statins for the treatment of solid cancer has been reviewed in this collection by Chimento et al. Statins prevent cancer growth and metastasis by interfering with Chol activities which include signal molecules on membrane rafts, substrate for steroids, oxysterols, and Vitamin D3 synthesis, ligand for estrogen-related receptor alpha (ERRα). This last aspect that proposes Chol as an endogenous ERRα agonist has been reviewed in depth by Casaburi et al. In several cancer cells, the expression and the activity of ERRα, together with its cofactors (PGC-1 α/β), is further influenced by oncogenic signals and can thus be re-directed to induce metabolic programs favoring tumor growth and progression (7). Based on these considerations, the use of therapeutic strategies aimed to reduce Chol levels, such as statins or drugs targeting the SREBP metabolic pathways, could be a promising option to counteract metabolic rewiring in cancer cells where ERRα plays a pivotal role. Another relevant signal molecule binding nuclear receptors and involved in cancer progression is the oxysterol 27-hydroxycholesterol (27HC). 27HC is an abundant Chol metabolite in the human circulation and promotes breast cancer cell proliferation (8, 9). Hiramitsu et al. in this collection clearly describe how this oxysterol promotes also lung cancer cell proliferation through Estrogen Receptor β/PI3K-Akt signaling. Thus, lowering 27HC levels may lead to a novel approach for the treatment of lung cancer. Oxysterols are also involved in the pathology of three major gastrointestinal cancer (hepatocellular carcinoma, pancreatic, and colon cancer). Kovac et al. in this collection suggest an interesting theory on the circadian clock effects on gastrointestinal carcinogenesis by regulating lipid metabolism and beyond. With this in mind, classical therapies (statins) to modulate Chol in gastrointestinal cancers, must be administered at a proper time and for a proper duration (chronotherapy) for successful therapies.

In conclusion, from this collection it emerges that regulation of sterol homeostasis is a complex network of transcription factors, protein modifiers, sterol transporters/carriers, sterol sensors disregulated in various pathological settings such as atheroscerosis, cancers, and neurodegenerative diseases. In this context Chol and oxysterol as signal molecules are an appealing target to counteract these pathologies. New strategies aimed to modulate their levels and actions in different tissues are worth to be developed.

## Author Contributions

RS, MU, and VP contributed conception and design of the study. All authors contributed to manuscript revision, read, and approved the submitted version.

### Conflict of Interest

The authors declare that the research was conducted in the absence of any commercial or financial relationships that could be construed as a potential conflict of interest.
